# Genome-Wide Identification and Functional Analysis of the Genes of the ATL Family in Maize during High-Temperature Stress in Maize

**DOI:** 10.3390/genes15081106

**Published:** 2024-08-22

**Authors:** Haiping Ding, Xiaohu Li, Shilin Zhuge, Jiyuan Du, Min Wu, Wenlong Li, Yujing Li, Haoran Ma, Peng Zhang, Xingyu Wang, Guihua Lv, Zhiming Zhang, Fazhan Qiu

**Affiliations:** 1 Hubei Hongshan Laboratory, National Key Laboratory of Crop Genetic Improvement, Huazhong Agricultural University, Wuhan 430070, China; dinghp@sdau.edu.cn (H.D.); lvgh@zaas.ac.cn (G.L.); 2 National Key Laboratory of Wheat Breeding, College of Life Sciences, Shandong Agricultural University, Taian 271018, China; lixiaohu814@163.com (X.L.); zgsl1997@gmail.com (S.Z.); dujiyuan814@163.com (J.D.); min1907778066@163.com (M.W.); 15615688160@163.com (W.L.); 17852407628@163.com (Y.L.); 13583893633@163.com (H.M.); zpeng0312@163.com (P.Z.); 13792691580@163.com (X.W.); 3 Zhejiang Academy of Agricultural Sciences, Institute of Maize and Featured Upland Crops, Hangzhou 310015, China

**Keywords:** maize, heat stress, *Arabidopsis*, abiotic stress, *Arabidopsis Toxicosa en Levadura*, gene family, tissue expression

## Abstract

Maize is a significant food and feed product, and abiotic stress significantly impacts its growth and development. *Arabidopsis Toxicosa en Levadura* (*ATL*), a member of the RING-H2 E3 subfamily, modulates various physiological processes and stress responses in *Arabidopsis*. However, the role of *ATL* in maize remains unexplored. In this study, we systematically identified the genes encoding *ATL* in the maize genome. The results showed that the maize *ATL* family consists of 77 members, all predicted to be located in the cell membrane and cytoplasm, with a highly conserved RING domain. Tissue-specific expression analysis revealed that the expression levels of *ATL* family genes were significantly different in different tissues. Examination of the abiotic stress data revealed that the expression levels of *ATL* genes fluctuated significantly under different stress conditions. To further understand the biological functions of maize ATL family genes under high-temperature stress, we studied the high-temperature phenotypes of the maize ZmATL family gene *ZmATL10* and its homologous gene *AtATL27* in *Arabidopsis*. The results showed that overexpression of the *ZmATL10* and *AtATL27* genes enhanced resistance to high-temperature stress.

## 1. Introduction

Maize (*Zea mays* L.) constitutes a significant portion of China’s three primary cereal crops and is also instrumental as animal feed and industrial raw materials [1,2]. Throughout its development, maize was often susceptible to a range of abiotic and biotic stresses, including drought, extreme salinity, heat, cold, and disease, which can inhibit corn growth and reduce crop yield [3,4]. Currently, during the maize growth process, heat damage and stem rot are the most serious abiotic and biotic stresses that significantly threaten grain yields. Stalk rot is a severe disease affecting corn. Infected plants develop soft lower internodes, leading to easy lodging and premature death during grain filling. This condition is primarily caused by Fusarium [5,6]. Plants have developed a range of intricate strategies to detect, react to, and adjust to harsh environmental conditions to ensure their survival. The regulation of genes is a crucial factor in the mechanisms through which plants manage both biotic and abiotic stresses [2,7]. Various transcription factors play a crucial role in plant responses to abiotic stress, including members of the *DREB* family, *WRKYs*, *MYBs*, and *bZIPs* [8,9]. Several phosphokinases are integral to abiotic stress, with all SnRK2s involved in heat stress and ABA signaling. The *TaSnRK2.11* gene in wheat responds to high-temperature stress [7,10]. The impact of stress goes beyond changes in membrane fluidity, calcium signaling, and MAPK activation, involving interactions among reactive oxygen species (ROS), nitric oxide (NO), phospholipid signaling, protein sumoylation, and 26S proteasome degradation [11,12,13]. Numerous studies have shown that ubiquitinated proteins play a crucial role in the responses to biotic and abiotic stresses in *Arabidopsis*. Nevertheless, there is limited understanding regarding the participation of ubiquitinated proteins in the responses to biotic and abiotic stresses in maize [14,15,16].

In eukaryotes, the ubiquitin (Ub)-directed protein degradation mechanisms oversee a variety of cellular functions, encompassing processes such as plant growth and morphogenesis, hormonal signaling, DNA repair, and responses to biotic and abiotic stressors [3,17]. E3 functions as a ubiquitin-protein ligase, facilitating the binding of Ub to the target protein by utilizing the E2 ubiquitin-conjugating enzyme in a sequential manner. This process imparts specificity for various substrates [18,19]. E3 ubiquitin ligases have become a pivotal element in the ubiquitination pathway, and they play a role in regulating plant responses to abiotic stress [18]. The literature indicates that ubiquitination commences with the activation of ubiquitin by an E1 enzyme (ubiquitin-activating enzyme), followed by the transfer of ubiquitin to an E2 enzyme (ubiquitin-conjugating enzyme), resulting in the formation of a thioester-linked E2 ubiquitin (E2-Ub) intermediate. It is hypothesized that the E3 enzyme recruited by the substrate (Ub ligase) may interact with E2-Ub, facilitating the transfer of ubiquitin to the target [20,21,22]. In the process of ubiquitination, E3 ligases play a crucial role in mediating the recruitment of specific target proteins, leading to their subsequent degradation [16,23]. Hence, there is a significant need to explore the functions of E3 ligases in response to biotic and abiotic stresses, particularly emphasizing their participation in heat stress.

In the realm of plant biology, three distinct categories of E3 ubiquitin ligases have been identified, namely RING, U-box, and HECT E3 ubiquitin ligases [24,25]. Among these, RING E3 ubiquitin ligases are prevalent in plants, with documented instances in maize indicating their role in abiotic stress responses [14]. E3 ligase *ZmAIRP4* is a direct homolog of *AtAIRP4*. *ZmAIRP4* functions as an active E3 ligase, and its overexpression has been shown to enhance drought tolerance in maize [26]. In maize, the E3 ubiquitin ligase *ZmRFP1* plays a role in conferring drought-stress tolerance in transgenic plants. This is achieved not only by improving water-retention capacity but also by enhancing the antioxidant system to mitigate ROS accumulation and reduce membrane damage [27]. In maize, two RING proteins, *ZmXerico1* and *ZmXerico2*, act as E3 ubiquitin ligases. Overexpressing *ZmXerico1* and *ZmXerico2* in maize improves water-utilization efficiency, thereby ameliorating yield performance under drought stress [28]. Although it has been established that E3 ubiquitin ligases play pivotal biological roles in abiotic stress responses that have been well-documented in Arabidopsis and rice (*Oryza sativa* L.), there is a lack of research literature focusing on this aspect in maize.

The *ATL* (*Arabidopsis Toxicosa en Levadura*) family containing a conserved RING-H2 domain in *Arabidopsis* also functions as E3 ubiquitin ligases to mediate plant responses to abiotic stress [29,30]. In yeast, *AthATL2* is the first member of the *ATL* family to be identified. Phenotypic characterization revealed that *AthATL2* expression is rapidly and transiently induced by pathogen-associated molecular patterns (PAMPs) [31,32,33]. In plants, the ATL protein family specifically targets proteins for degradation primarily via the ubiquitin–proteasome mechanism and serves as a pivotal regulator of plant resistance to abiotic stress [34,35]. For example, *OsATL2* and *OsATL38* act as negative regulators of cold tolerance in *O. sativa*, respectively [36]. In *Arabidopsis*, *AtATL78* has been demonstrated to fulfill disparate biological functions in response to drought and cold stress. *AtATL78* functions as a negative regulator of the cold stress response yet acts as a positive regulator of the drought-stress response [37,38]. In addition, *AtATL27* can improve salt resistance through alternative splicing under salt stress. The two alternative splicing factors interact with *CSN5A* and improve salt-stress resistance by regulating the expression of *CSN5A* [39]. Thus, the ATL family of proteins plays a critical role in the plant response to abiotic stress. Therefore, a better understanding of the functions of these unknown proteins and their molecular mechanisms may help develop more resistant crops that can withstand adverse environmental changes [32,40]. Correspondingly, the role of maize *ATL* family genes as E3 ubiquitin ligases in response to abiotic stress has yet to be elucidated.

Previous studies have shown that ATL genes play important biological functions under biotic and abiotic stresses in *Arabidopsis*, with similar findings reported in *O. sativa* [34]. However, the biological functions of ATL genes in *Z. mays* under such stresses remain to be elucidated. The aim of this study is to explore the family members of ATL genes in *Z. mays* and determine how ZmATL genes respond to abiotic and biotic stresses while understanding the role of ZmATL genes and their mechanisms in growth, development, and response to biotic and abiotic stresses. This study identified the members of the *Z. mays* E3 ubiquitin ligase ZmATL family by analyzing the whole genome and comprehensively examining the phylogenetic relationships and physicochemical properties of the 77 identified genes. Expression profiles of ZmATL genes under biotic and abiotic stresses revealed that ZmATL10 was upregulated by high temperature and could be induced by Fusarium verticillioides. Further phenotypic analysis under high temperatures confirmed that overexpression of ZmATL10 could enhance resistance to high-temperature stress.

## 2. Materials and Methods

### 2.1. Plant Material and Growth Conditions

Col-0 and *Atatl27* (SALK_034426) T-DNA insertion mutant alleles were used for all the experiments. The genotypes of *p35S*::*ZmATL10* and *p35S*::*AtATL27* were homozygous plants engineered via transgenesis. The seeds were sterilized for 6 h with chlorine before being placed on half-strength Murashigeand Skoog (½MS) medium (1.5% sucrose, 0.8% agar, and pH 5.8–5.85). The seedlings were grown in a growth chamber at 22 °C under a long-day photoperiod cycle (16 h light/8 h dark) for 1 week prior to immersion in stress conditions.

### 2.2. Plasmid Construction and Plant Transformation

For the p35S::ZmATL10-GFP construct, the CDS sequence of the *ZmATL10* coding region was cloned into the pBI121 vector, the CDS sequences of the coding region of ZmATL10 were cloned into the pBI121 vector, and the constructs were confirmed by sequencing. The primer pairs for vector ligation were: 35S::ZmATL10-FW,5-ctgcagtagacgcgtggatccATGAGGCCTCGCCGCCGG-3 and 35S::ZmATL10-RW, 5-gcccttgctcaccatggtaccTCACAATGGCAGCACCGAG-3. The specific primer pairs used for *AtATL27* were 35S::ZmATL27-FW, 5-ctgcagtagacgcgtggatccATGGTTATTATTCTCTGTCTCCCAT-3, and 35S::ZmATL27-RW, 5-gcccttgctcaccatggtaccTTATTATTCTCTGTCTCCCAT-3 from cDNAs of wild-type (WT) maize inbred line B73 and *Arabidopsis* Col-0 plant (WT), respectively. The PCR products were cloned into the pBI121 vector controlled by the cauliflower mosaic virus 35S promoter. After sequencing verification, the binary vector was introduced into *Agrobacterium tumefaciens* HA105. Wild-type (WT) plants were transformed by the floral dip method using strains containing *35S::ZmATL10* and *35S::AtATL27*, respectively. To select transgenic plants, T0 seeds were sterilized and screened in ½MS medium containing K-namycin to obtain positive plants. Then, the plants were transferred to a vermiculite medium for planting. After the individual plants were harvested, they were planted for verification. Finally, the T_2_ generation homozygous stable strains were obtained and confirmed by quantitative real-time (qRT)-PCR.

### 2.3. Identification of ZmATL Genes in Maize

The maize genome information, Zm-B73-REFERENCE-NAM- 5.0 (GCF_902167145.1), and the protein sequence of maize were retrieved from maizegdb (https://www.maizegdb.org/, accessed on 6 February 2023). The *Arabidopsis* ATL protein sequence was obtained from the TAIR database (https://www.arabidopsis.org/, accessed on 6 February 2023). The *Arabidopsis* ATL protein sequence was locally blasted against the maize dataset using TBtools software v1.112 (https://github.com/CJ-Chen/TBtools, accessed on 6 February 2023 [41]. A phylogenetic tree analysis of protein sequences of the ATL family in *O. sativa*, *Arabidopsis*, and *Z. mays* was performed using MEGA 11. The neighbor-joining method was selected (the bootstrap value was set to 1000). The iTOL (https://itol.embl.de/, accessed on 7 February 2023) online tool was then used to manipulate the phylogenetic tree [42]. MapInspect software v1.0 and TBtools (MCScanX) were used to analyze the chromosome location and colinearity of *ZmATL* family genes [41,43,44].

### 2.4. Analysis of the Physicochemical Properties of Maize ATL Family Proteins

To obtain the location information, MaizeGDB (https://www.maizegdb.org/, accessed on 6 May 2023) was utilized to retrieve the sequence length and amino acid details of the proteins encoded by the maize ATL gene family. The molecular weight and isoelectric point of the proteins encoded by the maize ATL family were analyzed using the bioinformatics tool ExPASy-Prot Param on ExPASy (http://web.expasy.org/protparam/, accessed on 6 May 2023). The Protter-interactive protein feature visualization tool (https://wlab.ethz.ch/protter/start/, accessed on 6 May 2023) was employed to examine transmembrane signals. Furthermore, the Plant-mPLoc tool (http://www.csbio.sjtu.edu.cn/bioinf/plant-multi/, accessed on 6 May 2023) was utilized to predict the subcellular localization of ATL family proteins.

### 2.5. Analysis of Gene Structure and Promoter Conserved Motifs

To detect conserved motifs in predicted RING-H2 finger proteins, the MEME tool with parameters set to an optimal width of 6–250 for each motif and a maximum number of motifs of 10 was used. In addition, motif annotation was performed using InterProScan (http://www.ebi.ac.uk/interpro/search/sequence-search, 7 February 2023) [45]. Maize gene structure GFF3 format files were downloaded from the Maize Genome Database (https://ensembl.gramene.org/Zea_mays/Info/Index, 7 February 2023). The systematic analysis of ATL family genes was performed using TBtools. Cis-regulatory elements within the promoter region (2000 bp upstream of the start codon) of ZmATLs were obtained online through PlantCARE (http://bioinformatics.psb.ugent.be/webtools/plantcare/html/, 6 May 2023). We then calculated the elements associated with growth and development, stress response, and hormone response.

### 2.6. High-Temperature, Drought-Stress, Salt-Stress Treatment

For high-temperature treatment, 7 days after germination, the seedlings were moved into a 42 °C incubator, and samples were taken at 0, 1, 3, and 6 h after treatment. For drought treatment, 300 mM mannitol were exogenously applied to the seedlings 7 days after germination, and samples were taken at 0, 6, 12, and 24 h after treatment. For salt stress, 250 mM NaCl were exogenously applied to the seedlings 7 days after germination, and samples were taken at 0, 6, 12, and 24 h after treatment. All collected samples were promptly frozen in liquid nitrogen for RNA extraction.

All *Arabidopsis* plants used in this study were of the Col-0 background. For heat-stress phenotype identification, seeds were sterilized with chlorine for 6 h before being placed on ½MS medium. The seedlings were grown at 22 °C for a week, then transferred to a 42 °C incubator for 2 h, and subsequently moved back to 22 °C for three days before being photographed. The control group was not subjected to heat treatment.

### 2.7. RNA Extraction and qRT-PCR Analysis

For the detection of *ZmATL* family genes through qRT-PCR, the total RNA was isolated from wild-type plants under high temperature, drought, and high salt stress and was extracted using TRIzol reagent (DP419 TIANGEN). cDNA was reverse transcribed using PrimeScript RT Reagent Kit and gDNA Eraser (Perfect Real-Time) Kit (RR047A; Takara Bio, Shiga, Japan). The RNA extraction and reverse transcription, as well as qRT-PCR, were performed as previously described [39]. The specific method was conducted using SYBR Green qPCR Premix (Low ROX). The two-step program comprised 95 °C for 5 min, 39 cycles of 95 °C for 30 s, and 60 °C for 60 s. Three technical replicates were conducted for each sample. The expression data were normalized using *18S* as an internal reference. Therefore, the primers used for qRT-PCR are shown in Table S1.

### 2.8. Abiotic Stress Responses in the ZmATL Family

To better understand the response of *ZmATL* family genes to abiotic stresses, such as high temperature, drought, and high salt, B73 (wild-type) maize seeds were sown in vermiculite and grown at 25 °C with a 16 h light/8 h dark photoperiod for 7 days. After two weeks of culture, the seedlings were subjected to abiotic stress treatments, including high temperature (42 °C), drought (250 mM mannitol), and high salt (300 mM NaCl), and the plants with normal watering were used as controls. Samples were collected at different time points after treatment for RNA extraction, and 3 biological replicates were performed for each treatment condition.

Then, the extracted RNA was quantitatively reverse-transcribed into cDNA, and then, a real-time quantitative PCR was performed to detect the expression levels of *ZmATL* family genes under different treatment conditions.

### 2.9. Biological Stress Responses in the ZmATL Family

To investigate the response of ZmATL family genes to Fusarium stalk rot (FSR), the sowed wild-type B73 maize seeds were planted in nutrient soil, cultured at 25 °C and a 16 h light/8 h dark cycle for 1 week, and then transferred to pots and placed them in a greenhouse at 25 °C and 16 h light/8 h to grow to the 5-leaf stage. The maize stalk rot pathogen (Fusarium verticillioides) was cultured on a PDA medium. Once the mycelium had developed, it was harvested and agitated on a mung bean medium for 48 h to induce spore production. The resulting bacterial suspension was then diluted to a concentration of 1 × 10^6^. Subsequently, 1 mL of the bacterial solution was injected into the 5-leaf stage maize stalks. Tissue samples from the infected site were collected at 12, 24, and 48 h post-infection and promptly frozen in liquid nitrogen for RNA extraction.

### 2.10. Tissue Expression Analysis of ZmATL Family Gene

To better understand the tissue expression level of the *ZmATL* family genes in maize, total RNA was extracted from the roots, stems, leaves, flowers, ears, and kernels of the maize inbred line B73 (wild type) during growth. Reverse transcription into cDNA was synthesized using the PrimeScript™ RT reagent kit (Takara, RR047A). Real-time quantitative PCR was then performed to detect the expression levels of *ZmATL* family genes across different tissues. Three technical replicates were performed for each sample. Expression data were normalized using *18S* as an internal reference. Therefore, the primers used for qRT-PCR are shown in Table S1.

### 2.11. Quantitation of Hydrogen Peroxide Radical

To measure the changes in hydrogen peroxide (H_2_O_2_) content after high-temperature stress treatment, WT, *35S::ZmATL10*, *35S::AtATL27*, and mutant *atl27* seedlings at 7 days after germination were treated with high temperature for 2 h. About 0.1 g of tissue was weighed using a hydrogen peroxide-content detection kit (Solebio, Beijing, China), and 1 mL of reagent was added for ice-bath homogenization. Centrifuged at 8000× *g* for 10 min at 4 °C, the resulting supernatant was taken, placed on ice, and then measured as described previously. The H_2_O_2_ content was determined by a previously reported method [39].

## 3. Results

### 3.1. Genome-Wide Identification of ZmATL Family Genes in Maize

**Figure 1:**
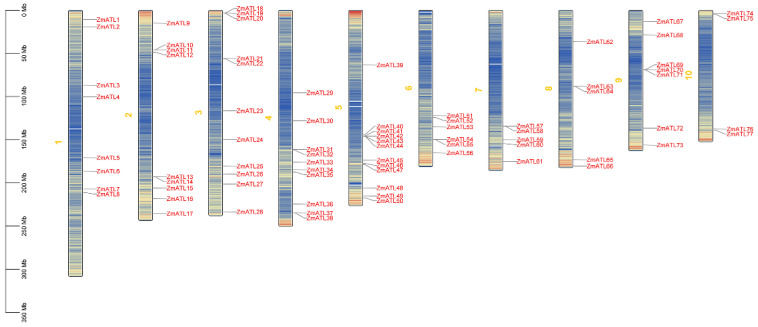
The chromosomal distribution of *ZmATL* family genes is illustrated in the figure. The red gene symbols denote various members of the *ZmATL* family. Maize chromosomes are indicated by yellow numbers. The density of genes is represented by the number of blue lines, with an increase indicating higher gene density and a decrease indicating lower gene density.

In the studies, amino acid sequences of the conserved Zinc/RING finger domain, C3HC4 (zinc finger) hit ATL in Z. *mays* are applied to identify homologs of ATL proteins in maize by using BLASTP (E-value < e^−5^) and the Markov hidden model (HMMER 3.2.1). Finally, a total of 77 members of the maize *ATL* family were identified and designated as *ZmATL1* to *ZmATL77* in accordance with their chromosomes. These members are extensively distributed across maize chromosomes. The *ZmATLs* names, gene ID, protein length, molecular weight (MW), theoretical isoelectric point (PI), and grand average of hydropathicity (GRAVY) are shown in Figure 1. The genomic sequence lengths of the *ATL* family genes vary widely, but the amino acid and transcript lengths are relatively stable, suggesting that the *ZmATL* family proteins are relatively conserved during evolution. The molecular weight ranged from 15,227 Da (*ZmATL37*) to 45,215.67 Da (*ZmATL51*), and the isoelectric point ranged from 4.81 (*ZmATL8*) to 11 (*ZmATL23*). Protein acid–base analysis showed that there were 38 acidic proteins and 39 basic proteins. The average hydrophilicity ranged between −0.438 (*ZmATL51*) and 0.591 (*ZmATL71*). The CDS length ranged from 438 bp to 1263 bp, and the aliphatic index ranged between 64.48 (*ZmATL75*) and 103.97 (*ZmATL71*) (Table 1). All of the *ZmATLs* contained conserved RING motifs and some highly conserved transmembrane structural domains (Figure S1). The RING motifs were well conserved. In *Arabidopsis*, proteins containing the RING domain usually play the function of E3 ubiquitin ligase. We selected several ATL proteins in *Z. mays* and *Arabidopsis* for conserved analysis and found that this family of genes is very conserved in *Arabidopsis*, *O. sativa*, and *Z. mays*.

### 3.2. Positioning Analysis of ZmATL Family Members

The objective of this study was to investigate the chromosomal distribution of the *ZmATL* family members on chromosome 10 in maize. To this end, the *ZmATLs* identified in the genome were located, and a chromosome distribution map was generated. The results demonstrated that the *ZmATLs* were distributed in a uniform manner across the 10 chromosomes of maize (Figure 1). *ZmATL* genes are widely distributed on different chromosomes, including chromosomes 3, 4, 5, and 6, which are clustered. This distribution pattern may be closely related to their important role in resisting abiotic stress. We also predicted the subcellular localization of *ZmATL* family members, and the results showed that most of the maize members were located in the cytoplasm and cell membrane. The results of intergenic colinearity analysis showed that 29 pairs of genes among the 77 *ZmATL* family members showed colinearity (Figure S2). Thus, the maize *ZmATL* gene family is extensively distributed throughout maize chromosomes, with the subcellular localization present in the cell membrane and cytoplasm.

### 3.3. Motif Composition and Gene Structure of ZmATL Genes

**Figure 2:**
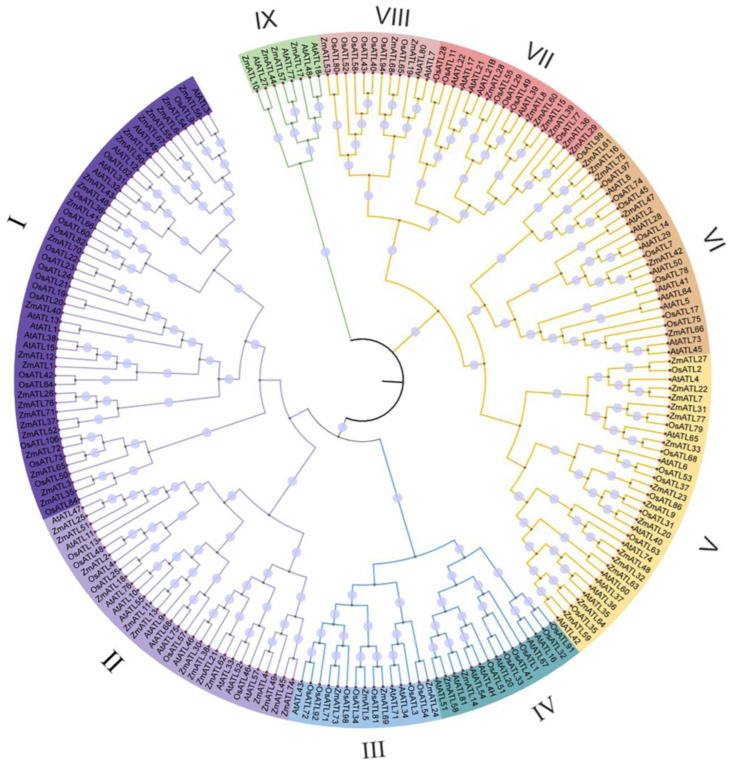
Phylogenetic analysis of ATL families of *A. thaliana*, *O. sativa*, and *Z. mays*. Roman numerals (I–IX) represent different family members, distinguished by different colors.

To explore the evolutionary lineage of ATL genes in plants, a phylogenetic analysis was performed on the ATL domain protein sequences derived from *Arabidopsis*, *O. sativa*, and *Z. mays*. According to the classification of *Arabidopsis AtATLs*, the *ATL* family can be divided into nine subfamilies, namely class I–class IX (Figure 2). All nine subfamilies contain family members from *A. thaliana*, *O. sativa*, and *Z. mays*, which indicates that the *ATL* family genes have a common ancestor.

**Figure 3:**
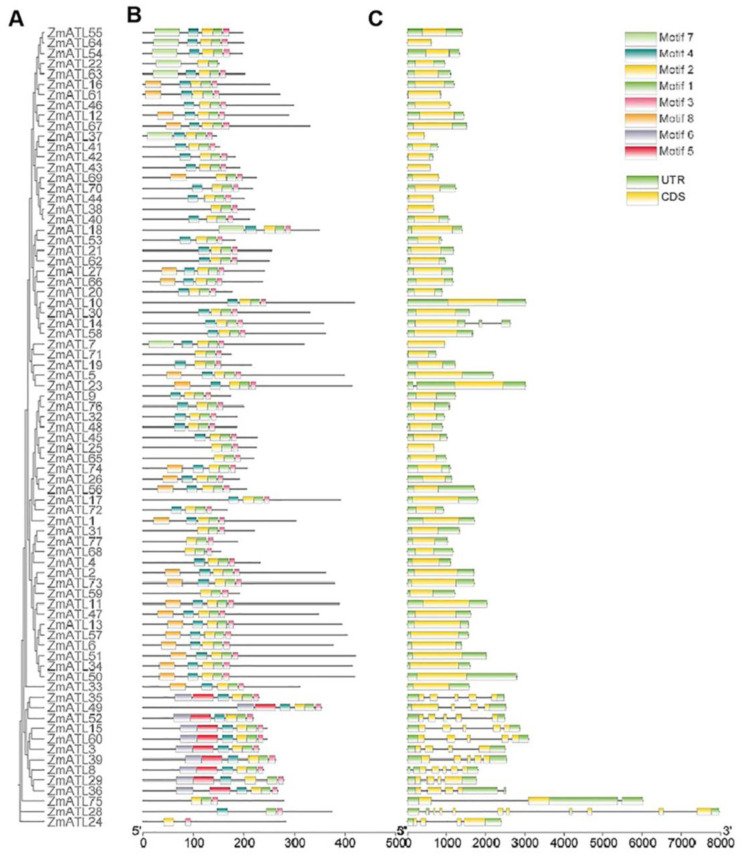
Phylogenetic tree, conserved motifs, and gene structure of maize ZmATL family. (**A**) Phylogenetic tree of ZmATL family members. (**B**) Distribution of conserved motifs in ZmATL proteins; colored boxes represent motifs 1–8. (**C**) Gene structure of ZmATL family genes, including introns (black lines), exons (yellow rectangles), and untranslated regions (UTRs, green rectangles).

The phylogenetic tree of the *ZmATL* family was obtained by the same method. The gene domain of the maize ATL family was drawn (Figure 3A). A bulk SMART search revealed that all ATL genes possess one to three RING finger domains, with certain *ATL* genes also containing regions from the Pfam:rad18 superfamily and HRD superfamily (Figure 3B). An analysis of the gene structure of *ZmATL* family members revealed that, out of the 77 family members examined, 60 lacked introns in their gene structure (Figure 3C). A subcellular localization analysis of maize ATL family proteins showed that most of its members were localized in the cell membrane and cytoplasm, which is consistent with the localization of ATL family members in *Arabidopsis*, which may be consistent with the function of RING domain proteins as E3 ubiquitin ligases. The results of a transmembrane domain analysis showed that maize ATL has multiple transmembrane domains, which is consistent with the results of conserved domain and transmembrane analysis.

### 3.4. ZmATLs Underwent Selection during Maize Domestication and Improvement

**Figure 4:**
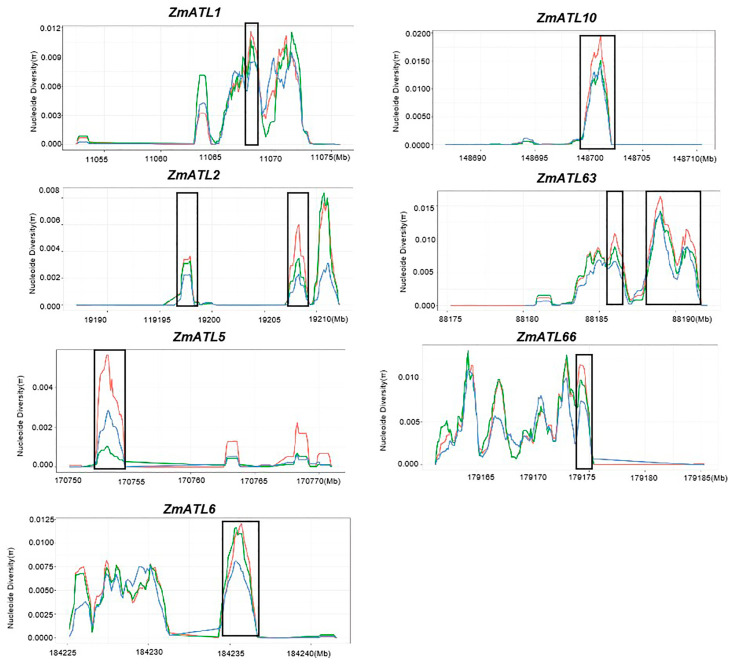
Evidence of selection pressure on *ZmATLs* is illustrated using maize HapMap v3 SNP data. The red, green, and blue lines indicate the nucleotide diversity of improved maize lines, landraces, and teosinte, respectively.

Maize represents, on average, only 57.1% of the nucleotide diversity in teosinte. Furthermore, a large fraction of maize genes has undergone domestication selection. The nucleotide mutation rate (π) serves as a significant indicator of selection in the process of domestication. To investigate whether *ZmATLs* have been selected during maize domestication and improvement, we calculated their nucleotide diversity in improved maize lines, landraces, and teosinte using HapMap 3. A nucleotide diversity analysis showed that *ZmATL3*, *ZmATL4*, *ZmATL18*, *ZmATL22*, *ZmATL52*, *ZmATL53*, *ZmATL54*, *ZmATL55*, and *ZmATL62* have undergone positive selection during maize domestication (Figure S3). However, *ZmATL1*, *ZmATL2*, *ZmATL5*, *ZmATL6*, *ZmATL10*, *ZmATL63*, and *ZmATL66* underwent negative selection throughout the maize domestication process (Figure 4). The significance of this choice still needs further exploration. Most of the remaining genes are not selected, and these results suggest that *ZmATL* genes are conserved in selective domestication.

### 3.5. Expression Patterns of the ZmATL Gene Family in Various Sugarcane Tissues

**Figure 5:**
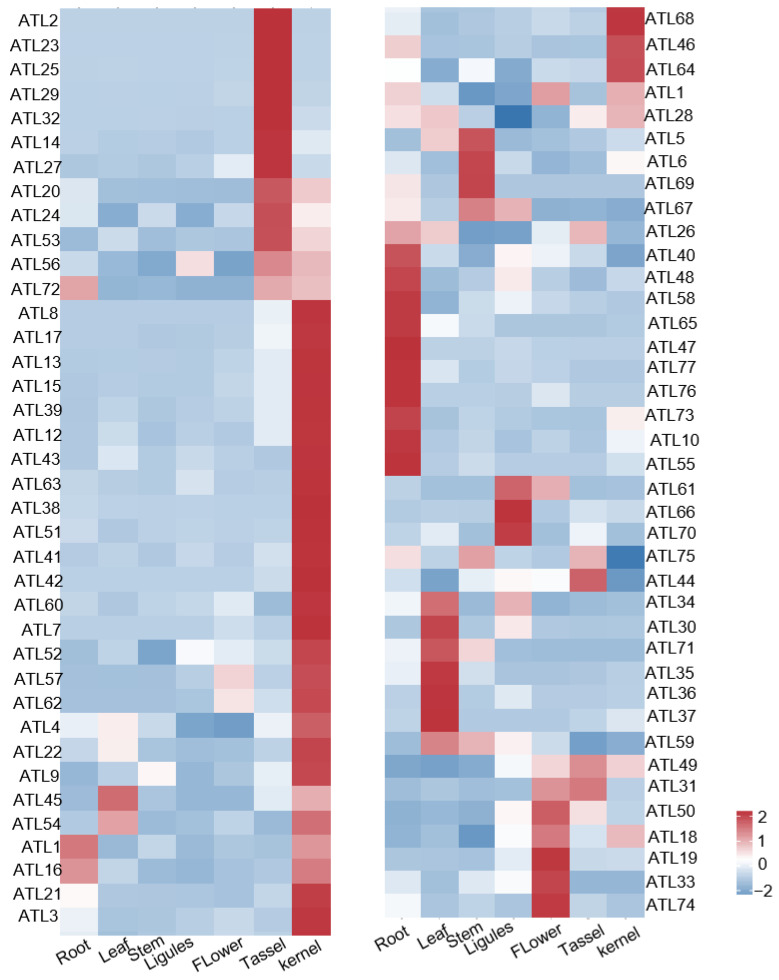
Heat map of tissue expression of maize *ATL* family genes.

The biological functions of maize ZmATL genes were investigated through an analysis of the expression profiles of the maize ZmATL gene family. We detected the tissue expression patterns of *ZmATL* genes in roots, leaves, young stems, ligules flowers, tassels, and kernels 9 days after pollination. The results showed that 77 family members were specifically expressed in different tissues. Among the 77 genes, 33 genes were found in all stages of maize, especially in kernels 9 days after pollination, such as *ZmATL8*, *ZmATL17*, *ZmATL13*, *ZmATL15*, *ZmATL39*, *ZmATL12*, *ZmATL43*, *ZmATL63*, *ZmATL38*, *ZmATL51*, *ZmATL41*, *ZmATL42*, *ZmATL60,* and *ZmATL7*. Additionally, it was discovered that ZmATL family genes, particularly *ZmATL50*, *ZmATL18*, *ZmATL19*, *ZmATL33*, and *ZmATL74*, were highly expressed in flowers. Genes *ZmATL2*, *ZmATL23*, *ZmATL25*, *ZmATL29*, *ZmATL32*, *ZmATL14*, *ZmATL27*, and *ZmATL20* were highly expressed in the tassel. *ZmATL26*, *ZmATL40*, *ZmATL48*, ZmATL58, *ZmATL65*, *ZmATL47*, *ZmATL77*, *ZmATL76*, *ZmATL73*, *ZmATL10*, and *ZmATL55* were highly expressed in roots. *ZmATL34*, *ZmATL30*, *ZmATL71*, *ZmATL35*, *ZmATL36*, *ZmATL37*, and *ZmATL59* are highly expressed in leaves (Figure 5). Additionally, some *ZmATL* genes were highly expressed ligules, such as *ZmATL61*, *ZmATL66*, and *ZmATL70*. *ZmATL5*, *ZmATL6*, *ZmATL69*, and *ZmATL67* were highly expressed in the stem. The expression patterns differed in various tissues, with 11, 7, 4, 3, 7, 12, and 33 genes having a higher expression in the root, leaf, stem, ligules, flowers, tassels, and kernels 9 days after pollination, respectively (Figure 5). The expression levels of ZmATL family genes are higher in young tissues. Based on this observation, we speculate that these genes may be closely related to stress response, as well as plant growth and development.

### 3.6. cis-Element Analysis of the ZmATL Genes in Maize

**Figure 6:**
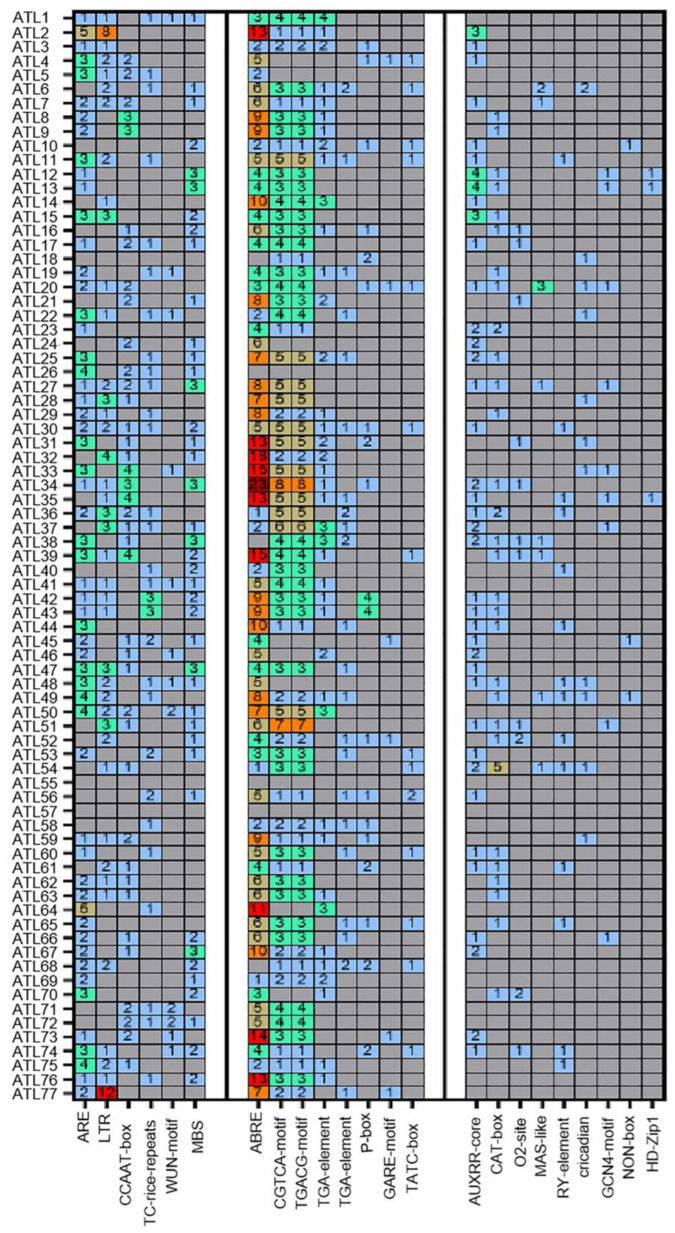
The promoter cis-regulatory elements of the *ZmATL* gene family are shown, with numbers representing the quantity of each element present in the promoter, where the numbers represent the number of contained elements, red represents more than 10 of these elements, orange represents 5–10 of these elements, and the rest represent less than 5 of these elements.

In order to study the ZmATL family genes and their potential biological functions, the 2000 base pairs of genomic sequences upstream of the ZmATL genes were obtained. Potential common cis-elements in the ZmATL promoter region were scanned using PlantCARE, followed by an analysis of the biological functions of these elements. The results revealed that several cis-elements are shared among *ZmATLs* (Figure 6). The cis-elements in the promoters of *ZmATLs* were identified and classified into three major groups, namely stress, phytohormone, and plant growth-related cis-elements. Among them, the ARE, LTR, CCAAT-BOX, TC-RICH, WUN-MOTIF, and MBS elements are important elements in stress response. ABRE, CGTCA, TGACG, TGA, P, GARE, TATC, and AuxRR are important elements of hormone response. At the same time, significant enrichment was found in the promoters of the ZmATL gene family, especially the three components ABRE, CGTCA, and TGACG. CAT, O2, MSA, RY, circadian, GCN4, NON, HD, and Motif I are important response elements for growth and development, but the enrichment of these elements in *ZmATL* promoters is significantly lower than that in hormone response elements.

Here, the results indicate that most *ZmATLs* contain abundant cis-elements that are responsive to hormones and stress. Notably, ABRE, a key cis-acting element involved in ABA-responsive gene expression, is significantly enriched in the promoters of *ZmATL* family genes. The CGTCA motif and TGACG motif are methyl jasmonate (MeJA) response elements. There is also a significant enrichment in the promoters of the *ZmATL* family of genes. The ABA-signaling pathway and MeJA pathway are central to stress responses in plants [7,46,47]. It was also found that most *ZmATL* genes contain multiple stress-response elements, including ARE (essential for anaerobic induction), LTR (involved in low-temperature stress), and MBS (MYB binding site associated with drought stress) (Figure 6). Numerous studies suggest that plant *ATLs* play a role in abiotic stress regulation. These elements are present in most *ZmATL* promoters, indicating that *ZmATLs* are likely induced by ABA and MeJA and involved in their signaling pathways.

### 3.7. Abiotic and Biotic Stress Analysis of ZmATL Genes Family Members

**Figure 7:**
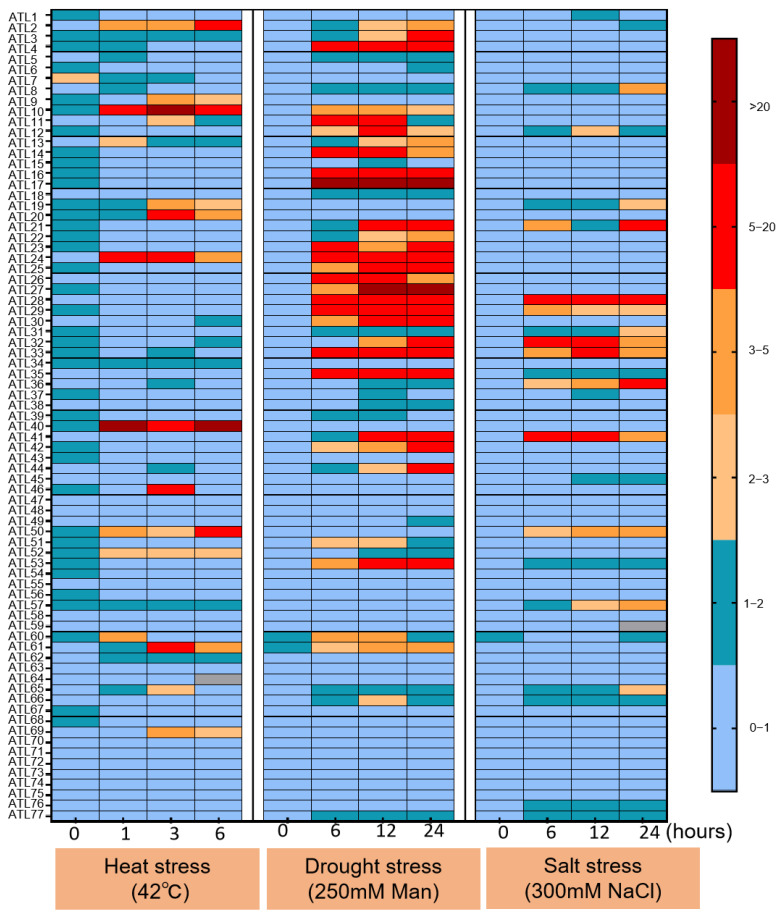
Abiotic stress heat map of maize *ATL* family genes. The expression at 0 h was set to 1. Color markers indicate changes in gene expression, red, orange, and pink for high expression and blue for low expression.

In order to study the response of *ZmATL* family gene expression to different forms of abiotic stress, the effects of high temperature, drought, and exogenous salt stress on the expression of *ZmATL* family genes were analyzed. One-week-old wild-type maize seedlings were treated with different stresses, including high temperature (42 °C), 250 mM mannitol, and 300 mM NaCl. The results showed that the expression values for *ZmATL10*, *ZmATL20*, and *ZmATL40* and the expression level were significantly up-regulated under high-temperature treatment. In contrast, *ZmATL1*, *ZmATL2*, *ZmATL22*, *ZmATL25*, *ZmATL27*, *ZmATL42*, *ZmATL49*, *ZmATL51*, and *ZmATL68* were obviously down-regulated after high-temperature treatment. Most of the genes were significantly up-regulated under drought stress, especially *ZmATL17*. Only a few genes were induced to be down-regulated, listed as *ZmATL43*. Under the condition of salt-stress treatment *ZmATL8*, *ZmATL12*, *ZmATL19*, *ZmATL21*, *ZmATL28*, *ZmATL29,* and *ZmATL32* were obviously induced to be up-regulated, but *ZmATL4*, *ZmATL24*, and *ZmATL73* were significantly down-regulated under drought stress. Under abiotic stress, the expression levels of maize *ATL* family genes varied significantly. Most genes responded to drought stress, while only *ZmATL10*, *ZmATL24*, and *ZmATL40* were significantly induced by high temperature (Figure 7).

**Figure 8:**
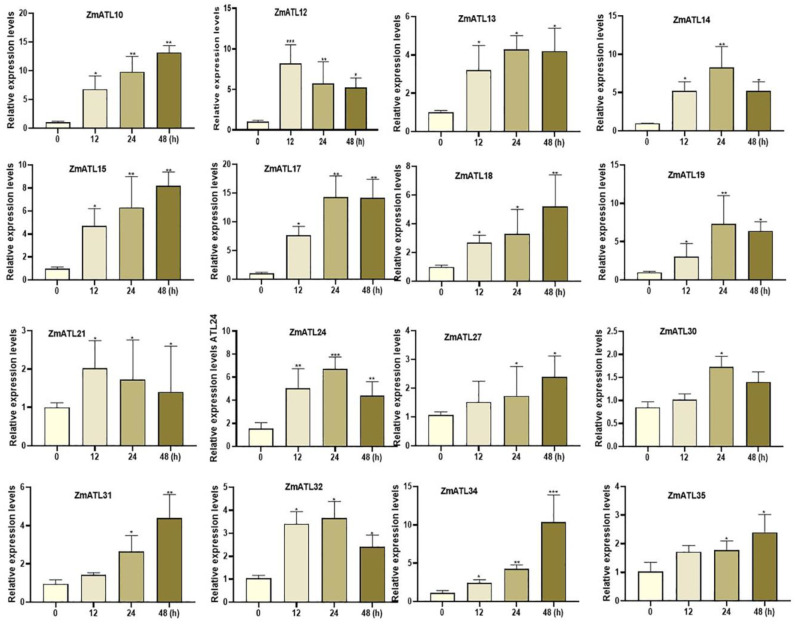
Expression levels of the *ZmATL* family genes following infection with *Fusarium stalk rot*. qRT-PCR was performed using gene-specific primers. These results show only genes that were induced and up-regulated(* *p* < 0.05, ** *p* < 0.01, *** *p* < 0.001).

To study the effect of maize *ZmATL* family genes on biotic stress, the effect of *Fusarium verticillioides* on the expression of *ZmATL* family genes was analyzed. Wild-type maize seedlings at the 5-leaf stage were used as research materials. Maize plants were infected with *F. verticillioides* for 12, 24, and 48 h, and the expression of Z*mATL* genes was analyzed. The results showed that *ZmATL10*, *ZmATL12*, *ZmATL13*, *ZmATL14*, *ZmATL15*, *ZmATL17*, *ZmATL18*, *ZmATL19*, *ZmATL21*, *ZmATL24*, *ZmATL27*, *ZmATL30*, *ZmATL31*, *ZmATL32*, *ZmATL34*, and *ZmATL35* were significantly induced. Among them, ATL12 and ATL21 were rapidly induced, and the other genes were induced at 24 and 48 h, respectively (Figure 8). Consequently, these genes are likely involved in resistance to *F. verticillioides*. We found that ZmATL10 was induced by heat and drought stress and was also involved in the response to *F. verticillioides*, indicating that ZmATL10 has a broad spectrum of stress resistance.

### 3.8. Functional Study of ZmATL10 and AtATL27 Resistance to High-Temperature Stress

**Figure 9:**
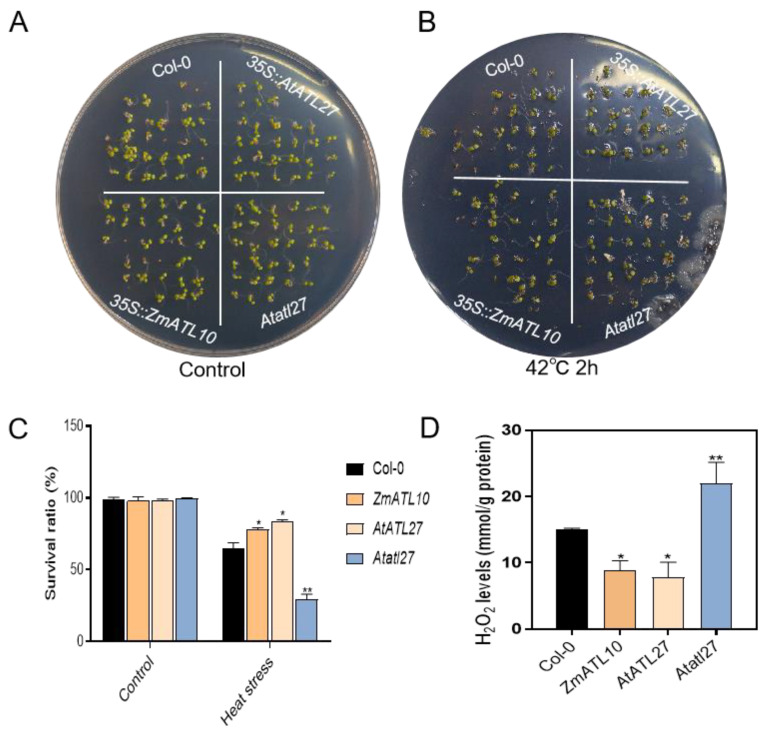
Phenotypes of Col-0, *ZmATL10*, *AtATL27*, and *Atatl27* seedlings after heat stress treatments. (**A**,**B**) Phenotypes of Col-0, *ZmATL10*, *AtATL27*, and *Atatl27* seedlings after 42 °C treatment. (**C**) Survival rate and hydrogen peroxide content in wild-type mutants treated with high temperature for 0 and 2 h. (**D**) Determination of hydrogen peroxide content in Col-0, *ZmATL10*, *AtATL27*, and *Atatl27* under high-temperature stress. Values are shown as the mean ± SE from three biological repeats. Statistically significant differences were identified between pairs of measurements using Student’s *t*-test (* *p* < 0.05, ** *p* < 0.01).

To further understand the biological function of *ZmATL10* under high-temperature stress, and also determine whether its homologous gene *AtATL27* in *Arabidopsis* also has similar functions, *35S::AtATL27* and *35S::ZmATL10* were used to construct overexpression transgenic lines in the Col-0 ecotype background by *Agrobacterium* inflorescence infection. Col-0, *Atatl27*, *35S::ZmATL10*, and *35S::AtATL27* overexpressed transgenic seeds were germinated in one-half MS medium for 7 days and then treated at 42 °C for 2 h. Compared with the survival rate of Col-0, the survival rate of *p35S::ZmATL10* and *p35S::AtATL27* was significantly higher than that of the wild type, while the survival rate of the *atl27* mutant was considerably lower than that of WT (Figure 9A–C). After high-temperature treatment, the hydrogen peroxide content of *35S::ZmATL10* and *35S::AtATL27* plants decreased compared with Col-0, while the hydrogen peroxide content of *Atatl27* mutant plants increased (Figure 9D). These results indicate that *ZmATL10* positively regulates high-temperature stress, which is consistent with the phenotype of its homologous gene *AtATL10* in *Arabidopsis*, indicating that the functions of maize *ATL* family genes are similar to those in *Arabidopsis*. Nucleotide diversity analysis showed that ZmATL10 underwent selection and domestication. The above results indicate that the functions of ATL genes in *Arabidopsis* and maize are relatively conserved, and they play the same function in resisting stress.

## 4. Discussion

In plants, the ATL subfamily is primarily characterized by the RING-H2 domain, which plays a crucial role in various metabolic processes related to plant growth and development [48]. Previous studies have shown that AtATLs contain a RING domain and multiple transmembrane domains and are thought to function as E3 ubiquitin ligases in *Arabidopsis* [49,50]. The number of ATL members varies across different plant species, with several articles reporting that there are 121 family members in rice (*O. sativa*), 96 in grapevine (*Vitis vinifera* L.), and 90 in poplar (*Populus trichocarpa*). There are 80 ATL members in *Arabidopsis*, 82 in tomato (*Solanum lycopersicum* L.), and 162 in soybean (*Glycine max* L.) [40]. However, the number of *ATL* family members in maize (*Z. mays*) and their expression patterns under abiotic stress have not yet been investigated. In this study, bioinformatics methods were employed to analyze the physicochemical properties, conserved domains, phylogeny, and expression patterns of maize ATL gene family members. In this study, 77 *ZmATL* genes were identified from the maize genome sequence, and they were evenly distributed across ten chromosomes. All *ZmATL* conserved domains contain only RING domains. Of these, 73 ZmATLs have transmembrane domains, while 4 ZmATLs do not (Figure S2). A phylogenetic analysis revealed that proteins from rice, *Arabidopsis*, and maize can be categorized into nine groups, each containing OsATLs, AtATLs, and ZmATLs. This suggests that ATL proteins from these three plants are closely related. In the evolutionary tree, the distribution of ZmATLs and OsATLs across branches is similar, indicating a closer relationship between maize and rice ATLs.

To create a comprehensive guide to the functions of ATL family ubiquitin ligases, we identified predicted ATL genes in *Arabidopsis*, *O. sativa,* and *Z. mays*. The identification of ATL family genes in *Arabidopsis* and *O. sativa* has been reported, and we further improved the functional study of maize *ATL* family genes [48,51,52]. The ATL family is a significant group of RING-H2 finger genes, comprising approximately 40% of the RING-H2 finger genes in maize. Previous articles reported that 80 ATL family genes were identified in *Arabidopsis* and 121 ATL genes were identified in *O. sativa* [48]. We used TBtools to identify 82 ATL genes in Arabidopsis, of which ATL4H and ATL28B were newly identified genes, which is different from previous reports. The results show that using TBtools in family gene mining can make changes more precise and convenient.

The ATL family has been reported to perform important biological functions as ubiquitin ligases in *Arabidopsis*. Ubiquitin ligase (E3) usually forms the ubiquitin–proteasome system (UPS) together with the ubiquitin-activating enzyme (E1) and ubiquitin-coupled enzyme (E2) [16,53]. The ATL family plays a crucial role in regulating a wide range of cellular processes, including protein degradation, signaling, DNA repair, cell-cycle progression, and immune response in *Arabidopsis* [39,40,48,51]. About 60% of rice *ATLs* are clustered with *Arabidopsis ATL* [48]. Similarly, we found through phylogenetic tree analysis that maize ATLs have high homology with ATLs in Arabidopsis and rice. According to the classification of *Arabidopsis AtATL*, the ATL family can be divided into nine subfamilies, namely class I–class IX. All nine subfamilies contain family members from *Arabidopsis*, *O. sativa*, and *Z. mays*. Gene structure analysis shows that about 78% of ATL family members in maize have no introns, which is consistent with the result that 90% of ATL genes in *Arabidopsis* lack introns. These genes have an ancient origin and may have appeared before the separation of monocots and dicots. Research on the *ZmATL* gene family in maize is currently lacking. However, its unique RING domain is a key marker of E3 ubiquitin ligases. Although the functions of the *ZmATL* family in maize remain unclear, preliminary evidence suggests that some members function in the same way as *Arabidopsis*, acting as RING-type E3 ubiquitin ligases involved in abiotic and biotic stress responses, hormone regulation, and plant growth and development, providing valuable references for future research.

As a sessile organism, maize cannot avoid abiotic stress during its growth and must withstand conditions such as soil salinity, drought, and extreme temperatures. These stresses significantly limit plant distribution, affect growth and development, and decrease crop productivity [54,55]. Abiotic stress can directly induce physical or chemical changes in plants at the physiological, biochemical, and cellular levels, triggering cellular stress responses. Disrupting sensor function affects the levels of second messengers, including Ca^2^⁺, ROS, No, and phospholipids [37,56,57]. To better adapt to environmental changes, maize has evolved an adaptive mechanism. A previous study showed that maize represents, on average, only 57.1% of the nucleotide diversity in teosinte [58]. The selective domestication analysis showed that most of the *ATL* genes were positively selected during evolution, indicating that ATL family genes were more suitable for the growth and reproduction of maize than other genes, and could improve the adaptability of maize to the environment. These results support the idea that multiple members of the gene family work together to regulate plant-specific traits. Selective domestication suggests that external stimuli, such as abiotic and biotic stresses, favor a wider abundance of this gene family, which helps this family better resist external stimuli. About 60% of the maize ATLs are clustered with *O. sativa* ATLs. Many of these predicted gene products show sequence similarity, indicating that they may be orthologous and may play the same biological function in plants.

In *Arabidopsis*, among the ATL family members, *AtATL2* was the first to be discovered. Initially, it was reported that overexpression of *AtATL2* in *A. thaliana* and yeast could cause yeast cell death, leading to conditional toxicity. Subsequent studies have shown that *AtATL2* plays a role in plant defense against pathogens [32,33]. The ATL protein family plays a crucial role in various aspects of plant growth and development, including developmental processes and stress-response mechanisms [36,49,50]. Several members of the ATL family have been functionally characterized, with some participating in plant defense [40]. For example, overexpression of *AtATL1* in *A. thaliana* results in growth retardation, characterized by stunted growth and cell death. Reducing *AtATL1* expression decreases the susceptibility of *A. thaliana* to powdery mildew [59]. The *Arabidopsis AtATL2* gene is also implicated in pathogen defense [30,33]. *AtATL5* in *A. thaliana* affects seed lifespan-related genes by regulating ABT1-mediated transcriptional activation [60]. The absence of ATL6 or ATL31/CNI1 increases the sensitivity to C/N stress; it is involved in regulating the growth process of *A. thaliana* [49,61,62]. AtATL27, as an E3 ubiquitin ligase, interacts with CSN5A and participates in plant salt-stress response to balance plant stress response and growth and development [39]. *Arabidopsis* AtATL54 is an E3 ubiquitin ligase that promotes secondary cell-wall biosynthesis and participates in programmed cell death during xylem formation [63]. AtATL62 is an ATL protein that is localized to the leaf epidermal plasma membrane, and it plays an important role in the regulation of flowering and photoperiod in plants [40]. ATL78 plays a dual role in *A. thaliana*, responding to both cold stress and drought stress. Overexpression of ATL78 can improve drought-stress resistance but is sensitive to cold stress [64].

In *O. sativa*, ATL family proteins also play important biological functions. For example, the ubiquitin ligase OsATL24 is crucial for promoting the development of meristem cells in roots. Additionally, the OsATL24 mutant exhibited increased sensitivity to nitrogen, which further inhibited lateral root formation. On the other hand, OsATL24 mutants exhibit heightened sensitivity to nitrogen, resulting in the inhibition of root formation [48]. The *ZmATL10* gene was overexpressed in *A. thaliana*, and phenotypic identification showed that the overexpressed plants showed an obvious high-temperature resistance phenotype. Continuing to study the response of ZmATL family genes to abiotic stress will help us continue to understand the functions of ZmATL family genes and cultivate new resistant varieties.

## 5. Conclusions

Plants experience various biotic and abiotic stresses during growth and development, leading to changes in their physiological, biochemical, cellular, and morphological characteristics. To cope with adverse growth conditions, plants regulate gene expression to respond to, adapt to, and resist stress. This regulation enhances their stress resistance. For instance, the ATL gene family in *A. thaliana* plays a crucial role in supporting growth and development under environmental stress. Thus, understanding the role of ATL is essential for comprehending nearly all aspects of plant growth and development.

In summary, the maize ATL family consists of 77 members, which are widely distributed in the cell membrane and cytoplasm. These members have highly conserved transmembrane transport domains and RING domains and show significant differences in expression levels across various tissues. They play crucial roles in resisting both biotic and abiotic stresses. We overexpressed the *ZmATL10* family genes from *Z. mays* in *Arabidopsis* and studied its homologous gene, *AtATL27,* in *Arabidopsis*. Overexpression of *ZmATL10* and *AtATL27* can significantly improve resistance to high-temperature stress.

The genes of the maize E3 ubiquitin ligase ZmATL family were comprehensively identified and systematically analyzed. This analysis revealed their expression patterns across various tissues and under both biotic and abiotic stresses. In summary, the biological function and evolutionary role of the maize ZmATL genes family under adversity have become clear. ZmATL genes are potential candidate genes for improving plant tolerance to biotic stress. In future studies, the role of ZmATL family genes in biotic and abiotic stress needs to be further explored, and the biological role of ZmATL family genes needs to be further clarified. Mining ZmATL family stress-resistant genes in maize and developing stress-resistant planting resources are of great significance to ensuring food quality and increasing food yield.

## Figures and Tables

**Table 1:** Physico-chemical properties of the Z*mATL* genes family in maize.

Gene Name	Gene ID	CDS (bp)	AA	MV (Da)	PI	GRAVY	Subcellular Localization
ZmATL1	Zm00001eb004000	1701	302	31,455.85	8.03	0.092	endosome
ZmATL2	Zm00001eb006670	1694	362	38,658.42	6.76	−0.349	membrane
ZmATL3	Zm00001eb022630	1279	230	23,747.31	6.76	0.425	membrane
ZmATL4	Zm00001eb024440	1093	232	24,741.12	7.19	−0.024	membrane
ZmATL5	Zm00001eb030780	2202	398	42,205.35	10.74	−0.125	membrane
ZmATL6	Zm00001eb033500	1383	377	40,128.52	9.04	−0.154	endosome
ZmATL7	Zm00001eb038810	960	319	33,829.16	7.19	−0.118	membrane
ZmATL8	Zm00001eb039970	1226	239	24,284.78	4.81	0.458	membrane
ZmATL9	Zm00001eb072750	1222	173	18,512.32	7.99	−0.07	membrane
ZmATL10	Zm00001eb081320	3022	419	43,467.62	6.73	−0.388	membrane
ZmATL11	Zm00001eb081330	2038	388	40,688.78	8.9	−0.301	membrane
ZmATL12	Zm00001eb081980	1442	289	29,534.54	8.71	−0.025	membrane
ZmATL13	Zm00001eb101180	1570	393	41,084.32	9.52	−0.197	membrane
ZmATL14	Zm00001eb101200	1739	357	38,217.32	5.9	−0.154	membrane
ZmATL15	Zm00001eb104970	1280	246	26,290.32	7.98	0.283	endosome
ZmATL16	Zm00001eb109400	1182	252	25,968.3	6.06	−0.129	endosome
ZmATL17	Zm00001eb115110	1791	390	40,387.1	9.26	−0.303	membrane
ZmATL18	Zm00001eb120050	1395	348	36,805.09	10.36	−0.052	membrane
ZmATL19	Zm00001eb120060	1212	214	22,058.23	8.32	0.176	membrane
ZmATL20	Zm00001eb120080	892	176	18,341.22	8.99	0.242	endosome
ZmATL21	Zm00001eb130410	1169	254	26,296.79	6.31	−0.07	endosome
ZmATL22	Zm00001eb130430	963	151	15,987.74	6.49	−0.187	endosome
ZmATL23	Zm00001eb135320	2923	414	43,420.32	11	−0.19	membrane
ZmATL24	Zm00001eb140640	1405	283	30,593.24	5.69	−0.417	membrane
ZmATL25	Zm00001eb146800	675	224	23,323.6	8.72	0.013	membrane
ZmATL26	Zm00001eb149500	1126	191	19,386.2	8.48	0.232	endosome
ZmATL27	Zm00001eb152830	1149	241	24,828.17	8.14	0.005	membrane
ZmATL28	Zm00001eb162790	1636	374	42,236	6.63	0.133	membrane
ZmATL29	Zm00001eb180470	1207	279	29,126.6	4.99	0.466	membrane
ZmATL30	Zm00001eb182770	1595	331	35,780.18	5.3	−0.343	membrane
ZmATL31	Zm00001eb187290	1326	220	23,625.18	5.39	0.092	endosome
ZmATL32	Zm00001eb187620	945	186	19,311.48	8.61	0.206	membrane
ZmATL33	Zm00001eb191210	1588	310	32,599.92	5.26	−0.123	endosome
ZmATL34	Zm00001eb193860	1610	415	45,209.22	9.49	−0.324	endosome
ZmATL35	Zm00001eb194620	1226	230	24,275.47	5.28	0.123	membrane
ZmATL36	Zm00001eb202890	1938	267	27,958.04	5.58	0.316	membrane
ZmATL37	Zm00001eb204430	441	146	15,227.8	8.78	0.589	membrane
ZmATL38	Zm00001eb204450	666	221	23,063.7	6.86	0.295	membrane
ZmATL39	Zm00001eb228180	1554	262	28,113.4	9.32	0.091	endosome
ZmATL40	Zm00001eb238220	1058	210	21,367.65	6.5	0.276	membrane
ZmATL41	Zm00001eb238400	776	152	15,603	8.03	0.363	membrane
ZmATL42	Zm00001eb238420	645	182	19,124.97	8.29	0.151	membrane
ZmATL43	Zm00001eb238430	579	192	19,995.74	6.87	0.114	membrane
ZmATL44	Zm00001eb238440	652	201	20,704.2	6.63	0.532	membrane
ZmATL45	Zm00001eb243170	1019	226	23,412.03	8.97	0.13	membrane
ZmATL46	Zm00001eb243960	1083	297	30,661.64	6.21	−0.041	membrane
ZmATL47	Zm00001eb244640	1630	347	36,916.78	5.85	−0.103	membrane
ZmATL48	Zm00001eb250960	894	185	19,146.14	8.45	0.189	membrane
ZmATL49	Zm00001eb254430	1586	353	38,628.32	10.24	−0.197	membrane
ZmATL50	Zm00001eb255510	2791	419	44,824.49	9.33	−0.289	endosome
ZmATL51	Zm00001eb279270	2018	421	45,215.67	5.73	−0.438	endosome
ZmATL52	Zm00001eb279770	1127	218	23,139.15	5.03	0.04	endosome
ZmATL53	Zm00001eb282260	869	182	19,018.83	5.07	0.324	endosome
ZmATL54	Zm00001eb285770	1322	198	20,760.37	6.29	−0.045	membrane
ZmATL55	Zm00001eb285790	1402	198	20,760.37	6.29	−0.073	membrane
ZmATL56	Zm00001eb290630	1701	206	20,530.3	6.12	0.211	membrane
ZmATL57	Zm00001eb315600	1565	404	42,765.13	9.01	−0.298	endosome
ZmATL58	Zm00001eb315620	1665	361	38,460.51	5.91	−0.174	membrane
ZmATL59	Zm00001eb319810	1206	191	19,657.09	5.09	−0.122	membrane
ZmATL60	Zm00001eb321320	1274	246	26,371.41	8.26	0.274	endosome
ZmATL61	Zm00001eb327570	858	271	28,116.63	4.98	−0.176	membrane
ZmATL62	Zm00001eb340160	970	251	25,820.13	6.07	−0.123	membrane
ZmATL63	Zm00001eb346450	1091	202	21,313.86	6.74	−0.199	membrane
ZmATL64	Zm00001eb346460	603	200	21,186.75	4.88	−0.009	endosome
ZmATL65	Zm00001eb366740	993	219	22,852.13	7.59	0.113	endosome
ZmATL66	Zm00001eb370730	1154	237	24,601.94	8.16	0.004	endosome
ZmATL67	Zm00001eb374170	1525	331	34,576.53	8.95	−0.045	membrane
ZmATL68	Zm00001eb379190	1152	154	16,678.46	8.46	−0.001	endosome
ZmATL69	Zm00001eb383450	797	224	23,890.18	8.51	0.391	endosome
ZmATL70	Zm00001eb383460	1227	216	22,714.36	6.35	0.317	membrane
ZmATL71	Zm00001eb383470	725	174	18,105.23	8.14	0.591	endosome
ZmATL72	Zm00001eb395020	924	166	17,299.07	7.54	0.446	membrane
ZmATL73	Zm00001eb401490	1696	380	40,216.01	6.16	−0.318	membrane
ZmATL74	Zm00001eb406080	1084	207	21,246.06	4.85	0.173	membrane
ZmATL75	Zm00001eb406570	3407	279	29,411.12	7	−0.314	endosome
ZmATL76	Zm00001eb428110	1070	200	20,678.66	8.49	−0.003	membrane
ZmATL77	Zm00001eb428450	1034	187	20,404.67	5.57	0.119	endosome

## Data Availability

All data are reported in this manuscript.

## Supplementary Materials

The following supporting information can be downloaded at https://www.mdpi.com/article/10.3390/genes15081106/s1: Figure S1: Analysis of transmembrane domains of *ZmATL* family genes. Figure S2: Synteny analysis of *ATL* family genes. Figure S3: Positive selection genes in the *ZmATL* family of genes during domestication. Table S1: Primers used in this study.
