# Supplementary material for: Genome-Wide Identification and Functional Analysis of the Genes of the ATL Family in Maize during High-Temperature Stress in Maize
Source: Genes (Basel). 2024 Aug 22;15(8):1106. doi: 10.3390/genes15081106 (PMC11353701; doi:10.3390/genes15081106)
Supplement: Supplementary file 1 [file genes-15-01106-s001.zip › Figure S.pptx]

## Slide 1
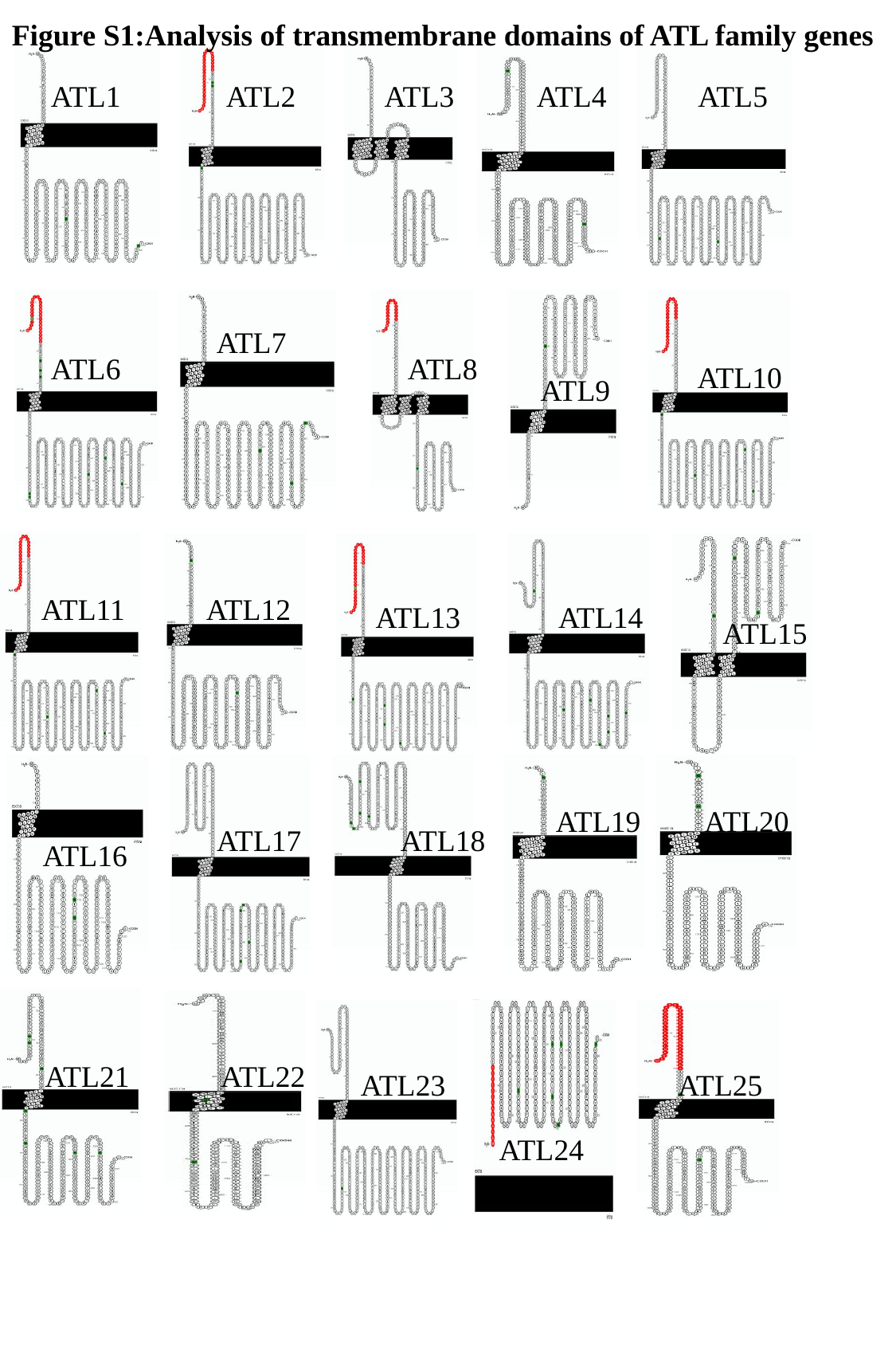

Figure S1:Analysis of transmembrane domains of ATL family genes
ATL1
ATL2
ATL3
ATL4
ATL5
ATL7
ATL6
ATL8
ATL10
ATL9
ATL11
ATL12
ATL13
ATL14
ATL15
ATL11
ATL19
ATL20
ATL17
ATL18
ATL16
ATL21
ATL22
ATL23
ATL25
ATL24

## Slide 2
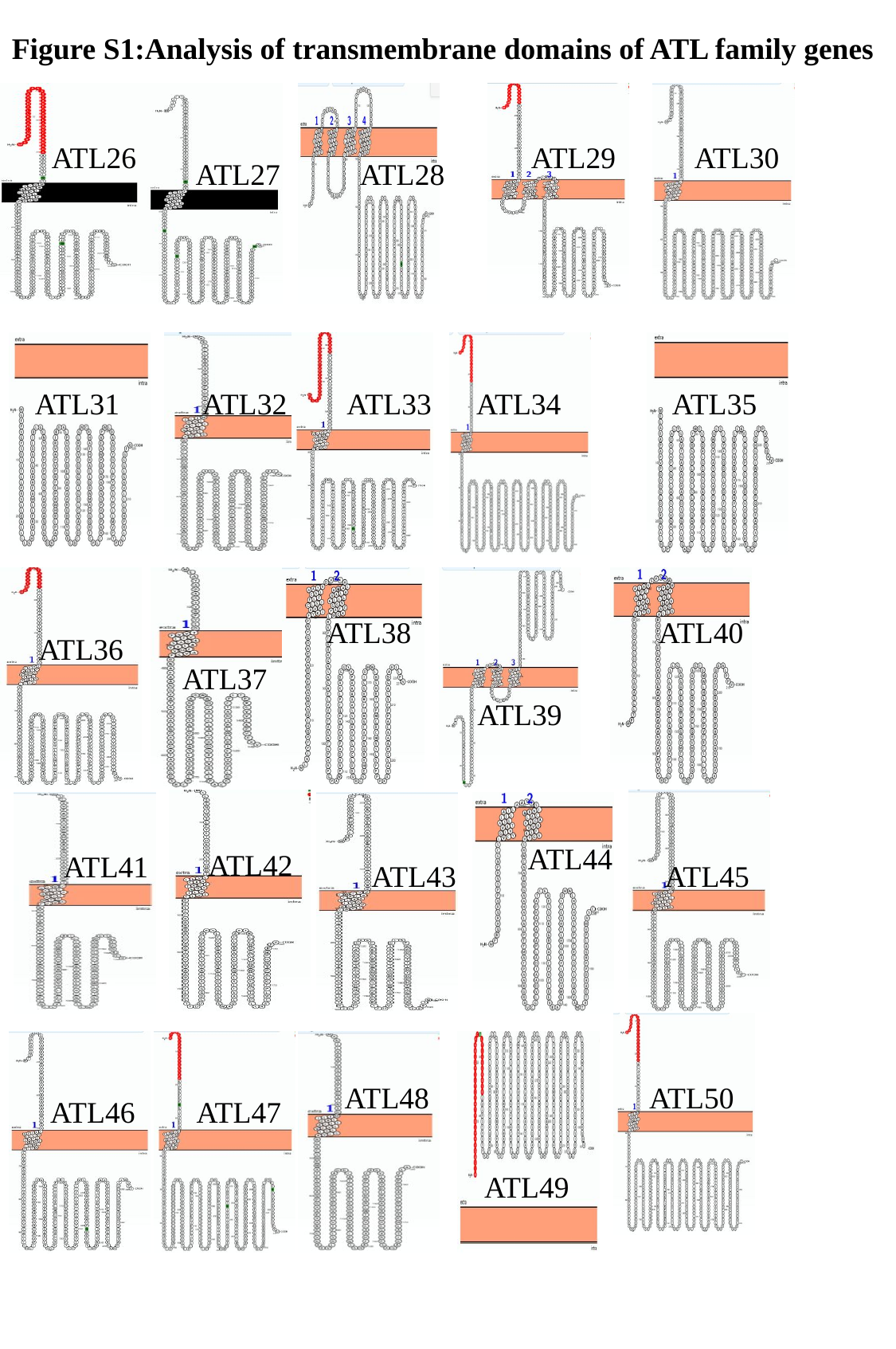

Figure S1:Analysis of transmembrane domains of ATL family genes
ATL26
ATL29
ATL30
ATL27
ATL28
ATL31
ATL32
ATL33
ATL34
ATL35
ATL38
ATL40
ATL36
ATL37
ATL39
ATL44
ATL42
ATL41
ATL43
ATL45
ATL48
ATL50
ATL46
ATL47
ATL49

## Slide 3
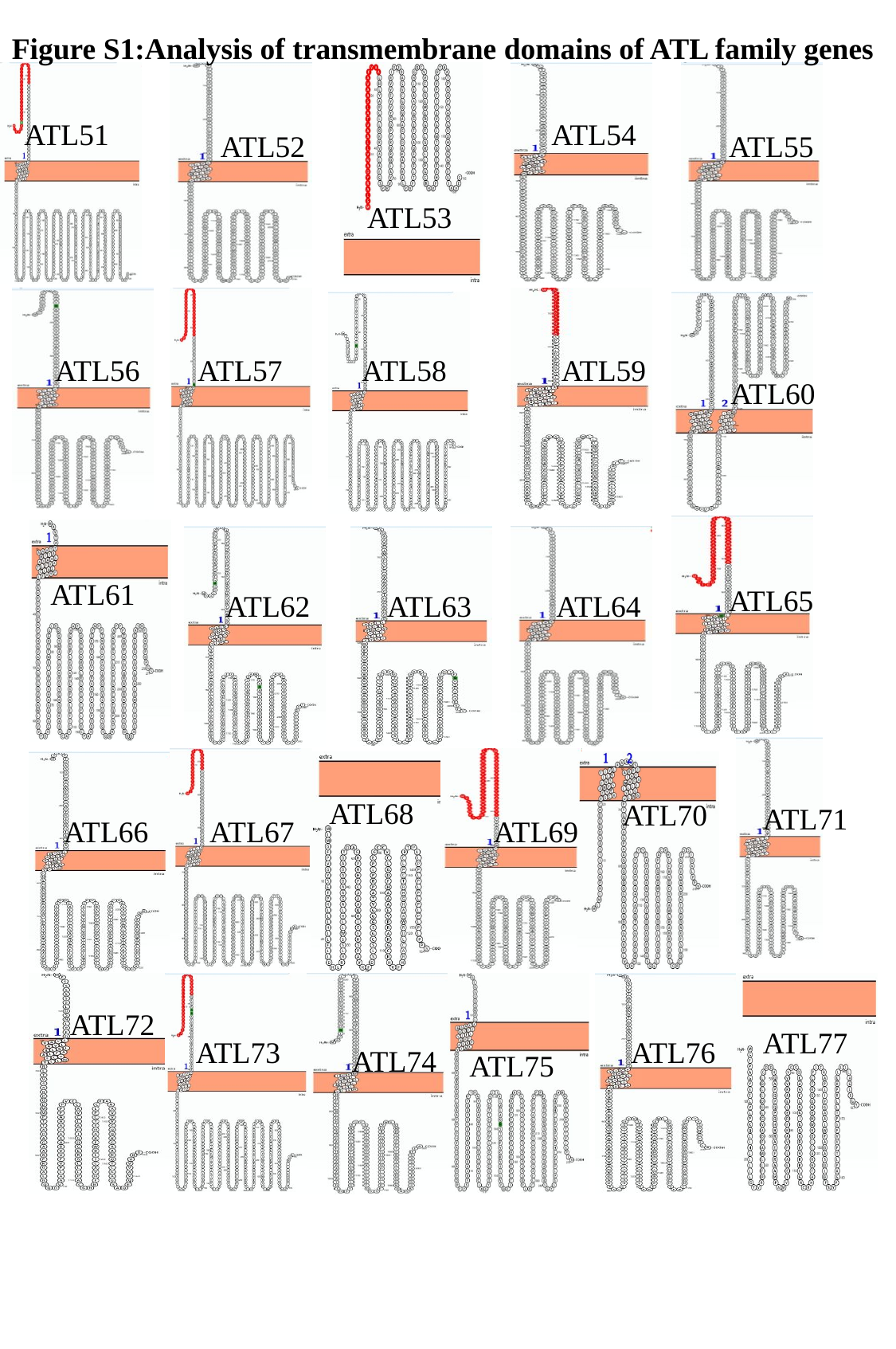

Figure S1:Analysis of transmembrane domains of ATL family genes
ATL51
ATL54
ATL52
ATL55
ATL53
ATL56
ATL57
ATL58
ATL59
ATL60
ATL61
ATL65
ATL62
ATL63
ATL64
ATL68
ATL70
ATL71
ATL67
ATL69
ATL66
ATL72
ATL77
ATL73
ATL76
ATL74
ATL75

## Slide 4
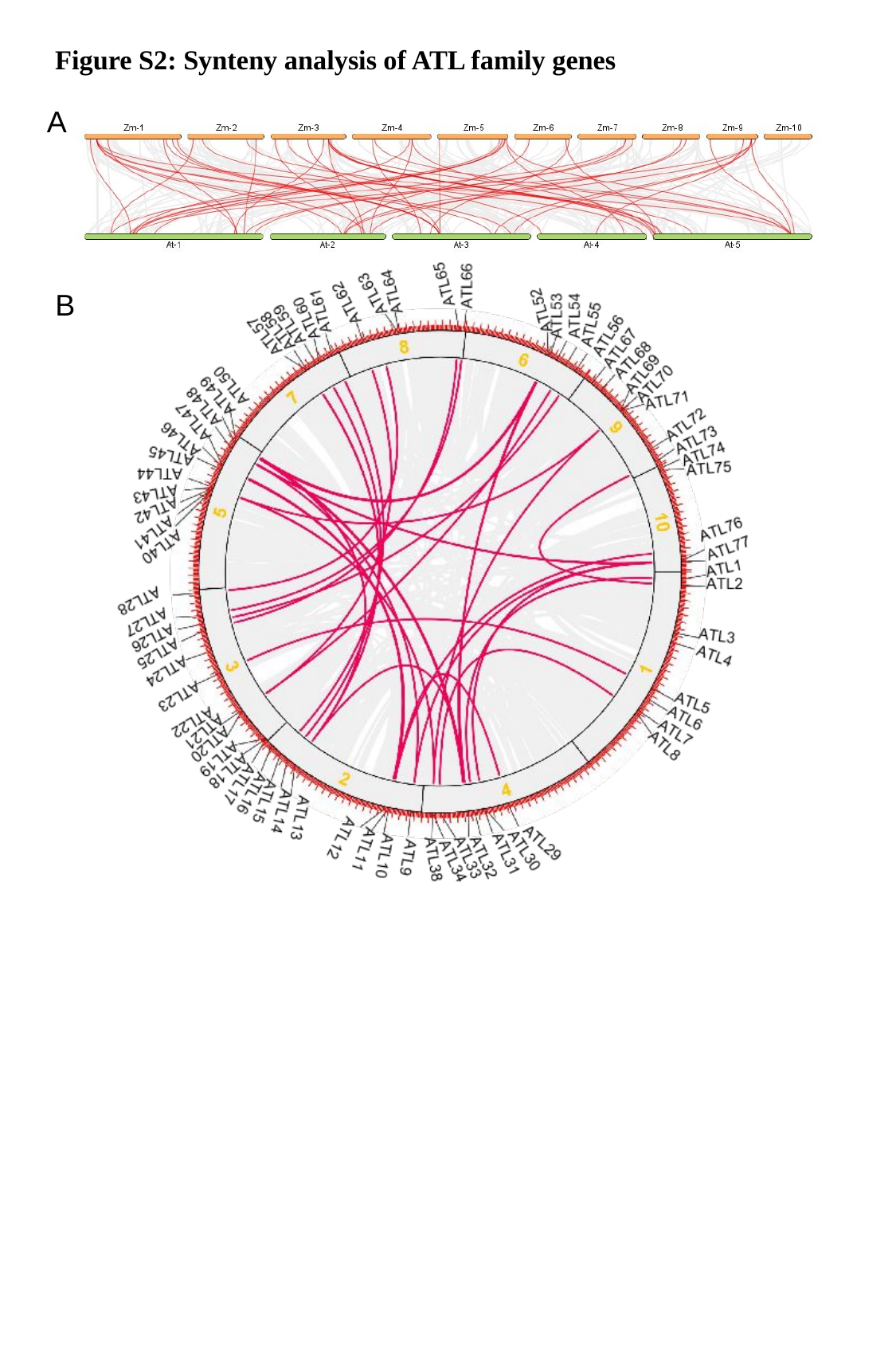

Figure S2: Synteny analysis of ATL family genes
A
B

## Slide 5
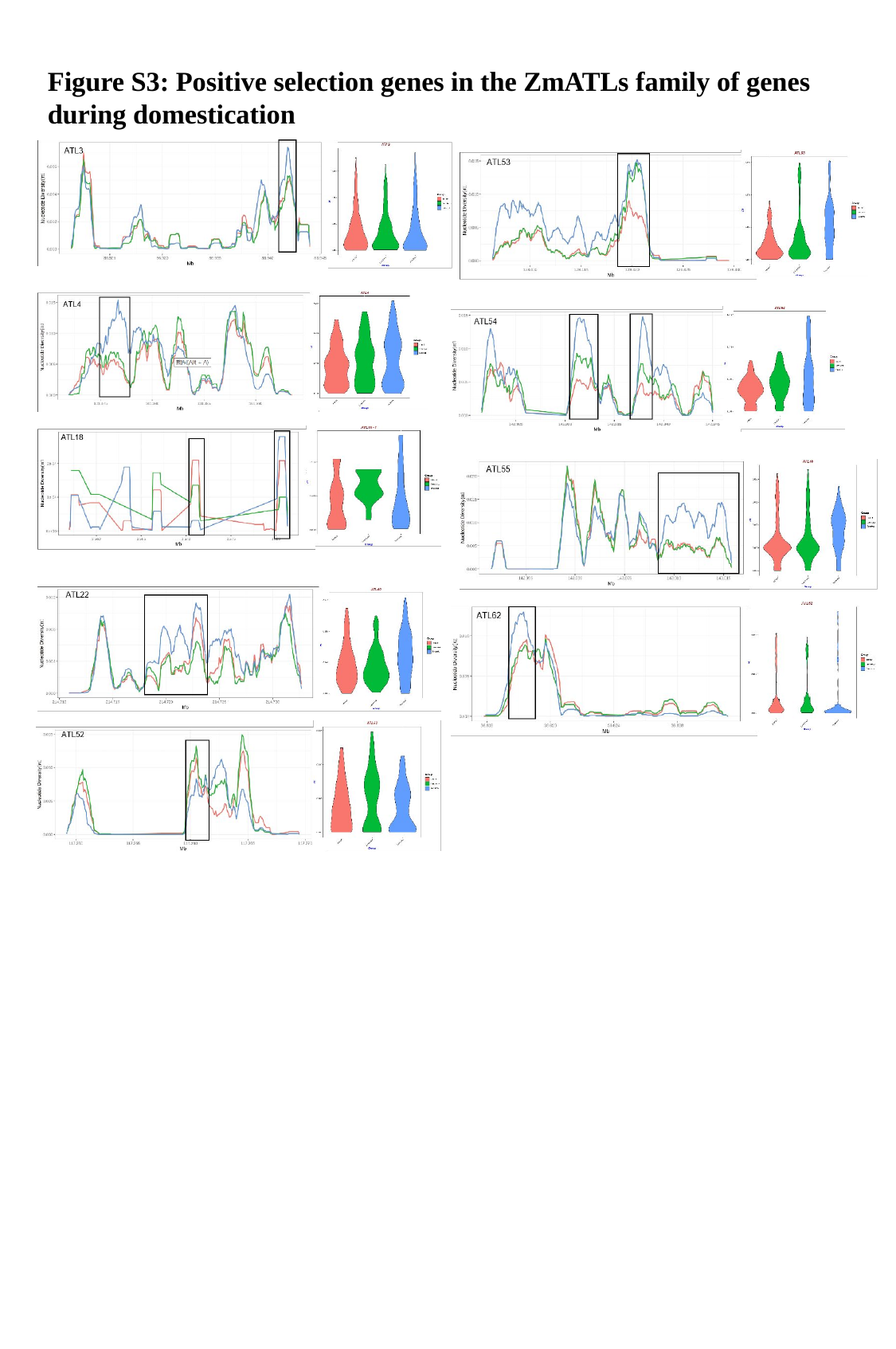

Figure S3: Positive selection genes in the ZmATLs family of genes during domestication
